# Feasibility and acceptability of a sleep health intervention among adolescents in Ugandan schools: A prospective pilot intervention study

**DOI:** 10.1093/sleepadvances/zpag029

**Published:** 2026-03-09

**Authors:** Denis Ndekezi, Rebecca Kyomugisha, Betty Nyangoma, Prossy Namirembe, Beatrice Nanyonga, Aaron Nyaruhuma, Claudia Ateo, Calvin Robert Rutainama, Katherine A Thomas, Ratifah Batuusa, Benson Muhindo, Sheilah Kasabiiti, Connie Alezuyo, Nambusi Kyegombe, Chris Bonell, Daniel Michelson, Fiona C Baker, Faith Orchard, Femke Bannink Mbazzi, Helen A Weiss

**Affiliations:** Department of Population Health, London School of Hygiene and Tropical Medicine, London, United Kingdom; Department of Social Determinants of Health, MRC/UVRI and LSHTM Uganda Research Unit, Entebbe, Uganda; Department of Social Determinants of Health, MRC/UVRI and LSHTM Uganda Research Unit, Entebbe, Uganda; Department of Social Determinants of Health, MRC/UVRI and LSHTM Uganda Research Unit, Entebbe, Uganda; Department of Social Determinants of Health, MRC/UVRI and LSHTM Uganda Research Unit, Entebbe, Uganda; Department of Social Determinants of Health, MRC/UVRI and LSHTM Uganda Research Unit, Entebbe, Uganda; Department of Research and Programs, Reach A Hand, Uganda; Department of Research and Programs, Reach A Hand, Uganda; Department of Research and Programs, Reach A Hand, Uganda; Department of Infectious Disease Epidemiology and International Health, International Statistics and Epidemiology Group, London School of Hygiene & Tropical Medicine, London, United Kingdom; Department of Social Determinants of Health, MRC/UVRI and LSHTM Uganda Research Unit, Entebbe, Uganda; Department of Research and Programs, Reach A Hand, Uganda; Department of Research and Programs, Reach A Hand, Uganda; Education Response Plan Secretariat, Ministry of Education and Sports, Uganda; Department of Social Determinants of Health, MRC/UVRI and LSHTM Uganda Research Unit, Entebbe, Uganda; Department of Global Health and Development, London School of Hygiene and Tropical Medicine, London, United Kingdom; Department of Public Health, Environments and Society, London School of Hygiene & Tropical Medicine, London, United Kingdom; Dept of Child and Adolescent Psychiatry, Institute of Psychiatry Psychology & Neuroscience, King’s College London, London, United Kingdom; NIHR Maudsley Biomedical Research Centre, South London and Maudsley NHS Foundation Trust and King’s College London, London, United Kingdom; Center for Health Sciences, SRI International, Menlo Park, CA, USA and School of Physiology, University of the Witwatersrand, Johannesburg, South Africa; School of Psychology, University of Sussex, Sussex, United Kingdom; Department of Population Health, London School of Hygiene and Tropical Medicine, London, United Kingdom; Department of Social Determinants of Health, MRC/UVRI and LSHTM Uganda Research Unit, Entebbe, Uganda; Department of Infectious Disease Epidemiology and International Health, International Statistics and Epidemiology Group, London School of Hygiene & Tropical Medicine, London, United Kingdom

**Keywords:** Sleep health, Insomnia, Cognitive behaviour therapy for insomnia, School-based intervention, Adolescents, Uganda

## Abstract

**Study Objectives:**

There is little research on sleep health interventions in Africa. We assessed the feasibility and acceptability of a tiered sleep health intervention among Ugandan adolescents. The intervention, delivered in two secondary schools, comprised universal components (sleep education sessions, structural changes to light, temperature and school-timings) plus targeted psychologist-delivered group cognitive behaviour therapy for insomnia (CBT-I) for students with moderate/severe insomnia (Insomnia Severity Index ≥15).

**Methods:**

Feasibility and acceptability were assessed through semi-structured interviews immediately after the intervention (T1) and 3 months later (T2) among students with baseline (T0) insomnia, teachers and dormitory matrons, along with structured implementation trackers. We conducted a quantitative survey at baseline to assess prevalence of dimensions of sleep and mental health, and for those with insomnia only, repeated this at T1 and T2.

**Results:**

The intervention was feasible and acceptable. High fidelity, dose and reach were achieved through integration of sleep education to the school schedule, structural changes to light, temperature and wake-up time for boarding students, effective small group delivery of CBT-I sessions and good retention despite fatigue due to extended sessions. Acceptability was reflected in high student engagement and positive feedback on the relevance of both universal and targeted components. Among 36 students with baseline insomnia, prevalence of moderate/severe insomnia decreased to 19.4% (7/36) post-intervention and further to 3.6% (1/28) at three months, indicating strong potential for impact.

**Conclusions:**

Multi-level, school-based sleep interventions can be successful in low-income settings. Large-scale cluster-randomized controlled trials are needed to estimate impact and cost-effectiveness.

**Statement of Significance**

Adolescent sleep problems are widespread in low-resource settings, yet evidence on the feasibility and acceptability of interventions to improve sleep health remains extremely limited. This study provides novel data that a school-based intervention delivering group cognitive behavior therapy for insomnia together with universal sleep education and structural changes can be delivered with high acceptability and may improve sleep and mental wellbeing among adolescents in Uganda. Based on the socio-ecological model of sleep health, the intervention addresses multi-component drivers of poor sleep that are often overlooked in similar contexts. These findings highlight the potential for scalable school-centred strategies to advance adolescent health. Further research is needed to robustly evaluate the effectiveness, cost-effectiveness and scalability of the intervention within national education systems.

## Introduction

Sleep health is critical for adolescents’ physical, cognitive, and emotional development [1]. There are multiple causes of poor sleep health in adolescents, aligning with a socio-ecological model (SEM) [2]. The SEM suggests that effective interventions to improve sleep health must address factors at individual level (e.g. knowledge, attitudes, practices), social (promoting a healthy sleep environment at home or boarding school), and societal (school start times, policy initiatives). Interventions at the individual level may be ineffective unless social and structural level factors are considered, and vice-versa [2]. This concept is supported by evidence that universal sleep education programmes alone in schools tend to be ineffective [3, 4], and it is recommended that universal approaches are combined with targeted components focused on changing behaviour and cognitive thinking among students with sleep disorders [4].

The most common sleep disorder among adolescents is insomnia, defined as frequent and persistent difficulty initiating or maintaining sleep despite adequate opportunity, and which results in daytime impairment [5]. Prevalence estimates of adolescent insomnia range from 4% to 39% [6], with bi-directional associations with depression and anxiety, poor academic performance, increased screen time, and other psychiatric disorders [6, 7]. Chronic insomnia can persist into adulthood, contributing to long-term health risks including cardiovascular disease, obesity, and poor mental health [6, 8].

The recommended first-line treatment for individuals with insomnia disorder, including adolescents, is cognitive-behavioural therapy for insomnia (CBT-I) [9]. CBT-I is a comprehensive structured treatment approach that includes sleep hygiene education, stimulus control, sleep restriction, cognitive strategies, and relaxation techniques [10]. Meta-analyses of randomized controlled trials (RCTs) among adolescents evaluating the impact of CBT-I have shown effectiveness on multiple dimensions of sleep health [9, 11, 12], as well as reductions in depression and anxiety symptoms [13–16]. However, effect sizes have generally been small, suggesting that while CBT-I is effective, the impact may be enhanced by complementary approaches such as sleep health education and structural or contextual adaptations.

Few studies have investigated efficacy of sleep interventions among adolescents living in African countries. A feasibility study of a group-based cognitive–behavioural sleep intervention among 61 adolescents with post-traumatic stress disorder in South Africa showed preliminary evidence of effectiveness on sleep quality [17]. A pilot cluster-RCT in Nigeria found locally-adapted group CBT-I to be feasible, with preliminary evidence of effectiveness on insomnia and depression among 50 adolescents [18]. These studies provide emerging evidence supporting the feasibility and potential effectiveness of CBT-I for adolescents in African contexts.

Uganda is home to one of the largest adolescent populations in Africa, both in absolute terms (9 million 10-19-year-olds) and as a proportion of its population (~25%) [19]. Poor sleep health is common among Ugandan adolescents [20, 21] and is associated with poor mental health and educational outcomes [21]. Contributing factors include demanding school schedules and poor awareness of the importance of sleep [20, 21]. In Uganda, an estimated 40% of secondary school students board [22], and environmental factors such as overcrowding and poor ventilation in dormitories negatively affect sleep quality and psychological wellbeing [23, 24].

Our objectives were to assess the (1) feasibility of implementing a tiered sleep health intervention in two Ugandan schools, focusing on fidelity, dose and reach (i.e. what was delivered and to what extent); (2) acceptability of the intervention among students with insomnia at baseline; and (3) indicative impacts of the intervention on dimensions of sleep health, dysfunctional beliefs about sleep, and mental health among participants with baseline insomnia.

## Materials and Methods

Design and setting: We conducted a pre-post intervention study in two private, mixed-sex secondary schools (Christian and Muslim) with both day and boarding students, in Wakiso district, Uganda, from August 2024 to February 2025. We used qualitative and quantitative methods to assess the feasibility, acceptability and initial impacts of a tiered sleep intervention. As this was a feasibility study, a formal sample size calculation was not conducted. The two schools were selected from participating schools with whom there were strong existing relationships due to participation in a recent school-based cluster RCT of a menstrual health intervention [25]. Inclusion criteria for schools were mixed-sex schools in Wakiso District documented as having ~40 Secondary 3 (equivalent to the third year of a four-year secondary Ordinary level education) female students based on MENISCUS trial data, and both day and boarding students. Of the four eligible schools in Wakiso district, one school was excluded as it was not within practical reach of the research base in Entebbe. The headteachers in the remaining three schools were contacted and asked to confirm their willingness for their school to participate. Two provided consent and were recruited. Both were privately owned (rather than Government). All students in Secondary 2 or 3 in May 2024 were eligible for participation.

Informed consent: We sought written informed consent from (1) parents or guardians of students aged less than 18 years, followed by electronic informed assent from students whose parents had consented. Parental/guardian consent was obtained primarily through in-person information meetings held at each participating school. For parents or guardians who were unable to attend these meetings, consent forms were sent home with students to request written authorization allowing the school headteacher to consent on their behalf for their child’s participation. (2) students aged *>*18 years; and (3) teachers, parents and district officials who participated in the qualitative interviews. Permissions covered all data collection and activities beyond the universal school-wide components, including participation in the targeted CBT-I sessions if students screened positive for insomnia.

Ethics approval: Ethics approval was granted by the Uganda Virus Research Institute Research & Ethics Committee (GC/127/819), the Uganda National Council of Science and Technology (HS1525ES), and the London School of Hygiene & Tropical Medicine interventional research ethics committee (22952).

Procedures: We conducted a baseline cross-sectional survey with students between July 7^th^ and August 23^rd^ 2024. Participants completed a questionnaire on tablets using Open Data Kit (ODK) software. In addition to socio-demographic and puberty development questions [26], we collected data on sleep health the full seven-item Insomnia Severity Index (ISI) [27] and five items selected to capture sleepiness from the 16-item Cleveland Adolescent Sleepiness Questionnaire (CASQ) [28] (Table S1). The total score of these five items has previously been shown to correlate strongly with the full CASQ score [29]. We assessed sleep-related cognitions (Dysfunctional Beliefs and Attitudes about Sleep (DBAS) [30, 31], and depression and anxiety symptoms using the 25-item UNICEF Measuring Mental Health Among Adolescents and Young People at the Population Level (MMAPP tool Domains 1 and 2; Table S2), which provide scores equivalent to the Patient Health Questionnaire-9 [PHQ-9] score for depression and Generalized Anxiety Disorder Assessment-7 [GAD-7] score for anxiety [32]. All survey tools were cognitively tested with a subset of students to assess appropriateness, clarity, and relevance, and adapted as needed. The 8-item Munich ChronoType Questionnaire (MCTQ) [33] was administered by trained researchers to minimize inaccuracy in these more complex responses on sleep patterns and timing (informed by cognitive testing).

Participants with moderate or severe insomnia (defined as an ISI score *>* 15 at baseline; hereafter called “insomnia”) were asked to repeat the baseline (T0) survey one week after completion of CBT-I (T1: November 2024) and 3 months after the end of CBT-I (T2: February 2025). Students without insomnia symptoms at baseline were not included in the follow-up survey, as the aim was to assess change in insomnia severity among those receiving CBT-I. The follow-up surveys included questions on perceptions of the intervention components.

To characterize the school environment and dormitory conditions, we conducted a structured transect walk in each school at baseline. During these walks, the research team, accompanied by school staff, systematically observed and documented features of the school compound, classrooms, and dormitories. Detailed observation notes were recorded to provide contextual information for understanding students’ sleep behaviours and potential environmental constraints.

Intervention: The intervention comprised universal and targeted components, described in accordance with the Template for Intervention Description and Replication checklist, a structured framework designed to improve the transparency and replicability of complex health interventions [34]. The universal component consisted of classroom-based sleep health education sessions and structural modifications (dim bulbs, fans, and school sleep/wake and teaching timing adjustments) aimed at improving the physical sleep environment and sleep routines within the schools (Table 1). These modifications were selected based on evidence that light exposure, temperature [35], and daily routines influence adolescent sleep quality [36]. These changes were made in consultation with stakeholders during a two-day stakeholder engagement workshop, ensuring feasibility, acceptability, and minimal disruption to school routines. Alternative strategies, such as changes to dormitory layouts or individual sleep aids, were considered but not selected due to logistical constraints and limited practicality within schools.

**Table 1:** TIDiER framework for the Better Sleep Better Health intervention among adolescents in Ugandan schools

**TIDiER item**	**Component 1: Sleep education sessions**	**Component 2: Structural changes**	**Component 3: Group CBT-I**
Why? Rationale	Improve healthy sleep behaviors by increasing awareness and understanding of sleep’s role in physical, mental, and academic well-being through school-based education.	Promote healthy sleep/wake patterns via adjusted school schedules (delay start times, reduce evening academic demands) and improved dormitory environment (light, ventilation).	Provide targeted, evidence-based support for adolescents with moderate–severe insomnia via culturally adapted group-based CBT-I to address cognitive and behavioral factors, tailored to the local school context.
What? Materials & Procedures	**Materials:** Facilitator manuals, presentation slides (tailored for students, teachers, parents), sleep awareness posters (age-appropriate illustrations), handouts summarizing key facts/strategies. **Procedures:** Interactive sessions for all S2 & 3 students and their parents, and all teaching staff. **Topics:** sleep cycles, importance for growth/cognition/mental well-being, strategies (consistent bedtimes, screen limits, relaxation). Posters displayed in classrooms/dormitories	**Materials:** Warm lightbulbs (7 W LED, 3500 K) & 16″ wall-mounted electric fans. **Procedures:** **Schedule changes:** Delay morning wakeup time; Delay morning class start-time at 8:00 a.m.; reduce evening prep time for boarders (allow earlier bedtimes); designate afternoon hours after 4:30 p.m. for peer interaction and homework. **Dormitory Improvements:** Install warm lightbulbs and fans to enhance ventilation & temperature regulation.	**Materials:** Context-specific facilitator manual, participant workbooks, daily sleep diaries (adapted from Consensus Sleep Diary). **Procedures:** Five weekly group sessions for students with ISI *>* 15. Led by clinical psychologists under senior clinical psychologist supervision. **Content:** Session 1: Introduction; setting rules, importance of sleep, sleep diary. Session 2: Physiology of sleep, insomnia and relaxation techniques. Session 3: Sleep hygiene, stimulus control and managing bedtime worries. Session 4: Sleep environment and Cognitive restructuring. Session 5: Maintenance of behaviour change and relapse prevention. Three groups conducted (one at one school; two at the other; one group size was 16). Participants completed daily sleep diaries for progress monitoring.
Who provided?	Trained peer educators, research team members.	Research team (schedule recommendations to headteachers); Qualified service provider (installations) supervised by research leader/headteacher.	Licensed clinical psychologists trained in adolescent mental health & CBT, supervised by a senior clinical psychologist.
How? Modes of Delivery	In-person interactive group presentations, discussions, and posters displayed in classrooms/halls (separately for students, teachers, parents).	Collaboration with school leadership for schedule changes. Physical installation of environmental modifications by qualified personnel.	5 weekly in-person group sessions in private school rooms. Structured manual, guided exercises, group discussion. Daily sleep diaries reviewed.
Where?	All intervention elements took place at the participating school campuses (classrooms, halls, dormitories, private rooms).		
When & how much?	Education: 1x session/group. Posters displayed throughout study	Implemented & maintained throughout study.	CBT-I: 5 weekly sessions (60-90 minutes each). Daily sleep diaries over 5 weeks.
Tailoring?	Not applicable		CBT-I adapted via stakeholder co-creation workshop to local school context/routines.
Modifications?	Not applicable		Minor content and scheduling adjustments during CBT-I based on participant feedback and school activities (e.g. exams). Documented.
How well?	Implementation tracker, fidelity checklists (education, CBT-I), and research team review meetings ensured protocol adherence. Reports on fidelity, dose, reach. Regular monitoring via diaries/feedback.		

The targeted CBT-I component was delivered to students who had screened positive for baseline insomnia, using a group format (10-16 students) led by trained clinical psychologists. We adapted the intervention from a Nigerian school-based group CBT-I intervention [18] through co-creation at the two-day stakeholder workshop. This ensured cultural relevance and age-appropriateness for Ugandan schools (Table S3). On the first day, clinical psychologists presented the proposed intervention and delivery plan to stakeholders, including parents, teachers, students, and district education and health officials. This was followed by discussions on how to implement the intervention in schools. On the second day, stakeholder feedback was used to develop a context-specific CBT-I facilitator manual.

Outcomes: We assessed the feasibility of implementing each intervention component with respect to fidelity, dose and reach (Table 2). We assessed fidelity using binary (yes/no) ratings of whether each intervention component was implemented as intended, using structured implementation tracking tools. Fidelity of structural changes was assessed through direct observation by the trained members of the research team using structured checklists to document whether planned modifications had been implemented, and to what extent. Observations were conducted by a pair of team members, and ratings were agreed upon through discussion at the time of assessment. Fidelity of the CBT-I component was assessed using structured checklists completed by the clinical psychologists delivering the intervention, documenting which activities were delivered, how, and with what fidelity. Dose was measured by tracking the number of activities delivered, including the total number of sleep education sessions and topics conducted for each group (teachers, students, parents); the number of structural installations completed in each school; and the detailed contents of CBT-I sessions delivered relative to the planned total. Reach was assessed by calculating the number and proportion of participants who engaged in each component, including teachers, students, and parents attending sleep education sessions; schools implementing sleep/wake adjustments and delayed start/end school time; and students with insomnia who participated in and completed CBT-I, and by the endline survey (Table 2).

**Table 2:** Data sources for fidelity (quality), dose delivery (completeness), reach (participation rate) feasibility and acceptability of the intervention components

**Intervention component**	**Fidelity**	**Dose**	**Reach**	**Feasibility and acceptability**
Sleep education sessions	Sleep education training reports Training attendance lists Implementation tracker Semi-structured interviews	Number of sessions delivered to students, teachers and parents Attendance list Implementation tracker Sem-structured interviews	Sleep education implementation tracker Implementation reports Semi-structured interviews	IDIs with the CBT-I students and Teachers
Structural changes (fans, lights and sleep/wake time)	Observation reports Installation report Implementation tracker	Number of fans and lights installed	Installation implementation tracker Implementation reports	IDIs with the CBT-I students and Teachers
CBT-I sessions	CBT-I training report Training attendance lists Implementation tracker CBT-I fidelity check list	Number of sessions delivered Time per session Attendance list	Attendance lists CBT-I implementation tracker Endline survey	IDIs with the CBT-I students Endline survey

We also investigated feasibility and acceptability through 12 semi-structured in-depth interviews (IDIs) conducted one week after the CBT-I intervention with eight CBT-I participants (three males, five females), two dormitory matrons, and two teachers (one male and one female per school). Follow-up IDIs were held at three weeks and three months post-intervention. Interviews, lasting 35–50 minutes, were conducted in English or Luganda and audio-recorded with participants’ consent. A topic guide covered experiences of whole-school components and CBT-I (where relevant), perceived usefulness of materials, and suggestions for improvement.

To assess the indicative impacts of the intervention among the sub-group of participants with baseline insomnia, we assessed pre-post scores on the ISI (insomnia), PHQ-9 (depression), and GAD-7 (anxiety), as well as changes in sleep timings and duration.

Data analysis: We analysed survey data in Stata version 18.0. Baseline characteristics for all participants were summarized by insomnia category (none, ISI *<* 7; subthreshold, ISI 8-14; or moderate/severe, ISI *>* 15). We used linear regression models to estimate mean differences and 95% confidence intervals (CIs) for the paired pre-post differences in ISI, PHQ-9 and GAD-7 scores between baseline and T1, and baseline and T2, respectively, adjusting for regression to the mean [37]. We used conditional logistic regression to analyse pre-post differences for binary and categorical data. We used two-sided tests to obtain *p*-values, except for the ISI outcome as participants were selected based on this measure; we hypothesized ISI score would decrease, partly due to regression to the mean [37]. Fidelity, dose, reach indicators and participant attendance were analysed descriptively.

Qualitative interviews were transcribed verbatim, translated into English when conducted in Luganda, and analysed using a codebook approach to thematic analysis [38]. Familiarization involved repeated reading of transcripts, followed by initial NVivo coding of four transcripts by two researchers independently to identify recurring patterns and refine the coding framework. A revised codebook was then systematically applied to all transcripts using an inductive approach to allow codes and themes to emerge from the data. Relevant sections were indexed with specific codes. Iterative analysis enabled the development of higher-level categories. Using axial coding, related codes were grouped into broader themes, and relationships between categories were explored. This enabled us to refine our understanding of patterns across participant groups in relation to the research questions. Coding discrepancies were resolved through discussion between the two coders, with final decisions reviewed by the wider research team to ensure consistency and credibility of the thematic analysis.

## Results

### Baseline sleep characteristics

Of the 366 students eligible for participation, 358 (97.8%) had written parental consent (if aged <18 years) and student assent, and participated in the study. The mean age was 15.8 years (standard deviation [SD] = 1.22, range 12-20 years). Overall, the reported median sleep duration was 5.1 hours (IQR 4.2-6.2). Compared with day students, boarding students reported shorter sleep duration (median 4.6 vs 6.3 hours; *p* < .001), more worry about sleep (30.1% vs 20.2%; *p* = .04) and greater dissatisfaction with sleep (77.7% vs 57.4%, *p* < .001).

The overall mean ISI score was 8.66 (SD 4.28), with 150 (41.9%) participants categorized as not having insomnia, 172 (48.0%) in the subthreshold range, and 36 (10.1%) as having moderate/severe insomnia. The 36 participants with insomnia were more likely than the others to be female, boarding students, in a larger household, to have eaten at least 2 meals the previous day and from a higher SES group (Table 3). They reported shorter sleep duration, less total time in bed (TIB), and poorer sleep efficiency (SE; duration/TIB) on school days than those without insomnia (mean hours sleep: 4.2 (SD = 1.7) vs 5.5 (SD = 2.3); mean hours TIB: 5.8 (SD = 1.4) vs 6.4 (SD = 1.7), SE *>* 85% (22.2% vs 69.3%)). Similar patterns were seen on non-school days (Table 3). There was weak evidence of a later chronotype (mid-point of sleep) among those with insomnia. Participants with insomnia were also more likely to report always having problems falling asleep (27.8% vs 6.7% among those with no insomnia symptoms). The most common reasons for having a poor night’s sleep in the participants with insomnia were the heavy school timetable (n = 11; 30.6%) and pain (n = 9; 25.0%). Most participants with insomnia (n = 32; 88.9%) had a DBAS score of *>*21 indicating substantial presence of dysfunctional beliefs and attitudes about sleep, compared with 58.0% of those without insomnia symptoms and 72.0% of those with subthreshold insomnia (Table 3).

**Table 3:** Characteristics of participants by insomnia score at baseline

	**Insomnia at baseline**			
	**No insomnia** **(ISI score *<* 7)**	**Subthreshold insomnia (ISI score 8-14)**	**Moderate/severe insomnia (ISI score *>* 15)**	***p*-value for association with ISI category**
**N (%)**	150 (41.9%)	172 (48.0%)	36 (10.1%)	
**School**				
Muslim	89 (59.3%)	100 (58.1%)	20 (55.6%)	0.92
Christian	61 (40.7%)	72 (41.9%)	16 (44.4%)	
**Age in years**	15.7 (1.2)	15.9 (1.2)	15.9 (1.4)	
**Age group (years)**				
<=15	58 (38.7%)	64 (37.2%)	16 (44.4%)	0.22
16	56 (37.3%)	54 (31.4%)	7 (19.4%)	
> = 17	36 (24.0%)	54 (31.4%)	13 (36.1%)	
**Sex**				
Female	75 (50.0%)	111 (64.5%)	27 (75.0%)	0.004
Male	75 (50.0%)	61 (35.5%)	9 (25.0%)	
**Religion**				
Christian	53 (35.3%)	62 (36.0%)	17 (47.2%)	0.26
Muslim	97 (64.7%)	108 (62.8%)	18 (50.0%)	
None/Other	0 (0.0%)	2 (1.2%)	1 (2.8%)	
**Day or Boarding**				
Day	65 (43.3%)	53 (30.8%)	11 (30.6%)	0.05
Boarding	85 (56.7%)	119 (69.2%)	25 (69.4%)	
**Household size**				
> = 8	59 (39.3%)	64 (37.2%)	20 (55.6%)	0.09
6-7	53 (35.3%)	49 (28.5%)	10 (27.8%)	
0-5	38 (25.3%)	59 (34.3%)	6 (16.7%)	
**Primary caregiver**				
Mother	82 (54.7%)	93 (54.1%)	23 (63.9%)	0.33
Father	50 (33.3%)	48 (27.9%)	7 (19.4%)	
Other	18 (12.0%)	31 (18.0%)	6 (16.7%)	
**Meals ate yesterday**				
≤1	10 (12.2%)	22 (22.4%)	0 (0.0%)	0.02
> = 2	72 (87.8%)	76 (77.6%)	22 (100.0%)	
**Socioeconomic status**				
Lowest	37 (24.7%)	33 (19.2%)	3 (8.3%)	0.01
Medium-low	30 (20.0%)	35 (20.3%)	6 (16.7%)	
Medium	23 (15.3%)	43 (25.0%)	7 (19.4%)	
Medium-high	24 (16.0%)	39 (22.7%)	7 (19.4%)	
Highest	36 (24.0%)	22 (12.8%)	13 (36.1%)	
**Sleep-related characteristics**				
**Sleep duration**				
Schooldays (mean (SD))	5.5 (2.3)	4.9 (1.5)	4.2 (1.7)	*p* < .001
<7 hours	115 (76.7%)	159 (92.4%)	35 (97.2%)	*p* < .001
7-11 hours	34 (22.7%)	13 (7.6%)	1 (2.8%)	
> = 12 hours	1 (0.7%)	0 (0.0%)	0 (0.0%)	
Non schooldays (mean (SD))	7.0 (2.5)	6.6 (2.5)	5.4 (2.7)	*p* = .003
<7 hours	79 (52.7%)	98 (57.0%)	27 (75.0%)	*p* = .15
7-11 hours	66 (44.0%)	71 (41.3%)	8 (22.2%)	
> = 12 hours	5 (3.3%)	3 (1.7%)	1 (2.8%)	
**Total time in bed**				
Schooldays (mean (SD))	6.4 (1.7)	6.0 (1.6)	5.8 (1.4)	0.06
<7 hours	94 (62.7%)	134 (77.9%)	29 (80.6%)	*p* = .02
7-11 hours	54 (36.0%)	38 (22.1%)	7 (19.4%)	
> = 12 hours	2 (1.3%)	0 (0.0%)	0 (0.0%)	
Non schooldays (mean (SD))	7.7 (2.5)	7.4 (2.6)	6.6 (2.6)	0.02
<7 hours	53 (35.3%)	70 (40.7%)	24 (66.7%)	*p* = .01
7-11 hours	89 (59.3%)	96 (55.8%)	10 (27.8%)	
> = 12 hours	8 (5.3%)	6 (3.5%)	2 (5.6%)	
**Sleep efficiency (school days)**				
*>*85% (good).	104 (69.3%)	86 (50.0%)	8 (22.2%)	*p* < .001
<85% (poor)	46 (30.7%)	86 (50.0%)	28 (77.8%)	
**Sleep efficiency (non-school days)**				
*>*85% (good)	119 (79.3%)	120 (69.8%)	19 (52.8%)	*p* = .001
<85% (poor)	31 (20.7%)	52 (30.2%)	17 (47.2%)	
**Chronotype**				
<3 a.m. (early)	59 (39.3%)	76 (44.2%)	9 (25.0%)	*p* = .10
> = 3 a.m. (late)	91 (60.7%)	96 (55.8%)	27 (75.0%)	
**During the past two weeks, how often have you had problems falling asleep**				
Never	50 (33.3%)	28 (16.3%)	3 (8.3%)	*p* < .001
Sometimes	80 (53.3%)	99 (57.6%)	20 (55.6%)	
Often	10 (6.7%)	20 (11.6%)	3 (8.3%)	
Always	10 (6.7%)	25 (14.5%)	10 (27.8%)	
**What is the most common reason you typically have a poor night sleep?**				
It is too noisy	30 (21.6%)	31 (18.0%)	5 (13.9%)	*p* < .001
It is to light	16 (11.5%)	5 (2.9%)	1 (2.8%)	
It is too hot/cold	24 (17.3%)	17 (9.9%)	4 (11.1%)	
I am worried or anxious	7 (5.0%)	33 (19.2%)	6 (16.7%)	
I am in pain	14 (10.1%)	19 (11.0%)	9 (25.0%)	
Heavy school timetable	41 (29.5%)	62 (36.0%)	11 (30.6%)	
Other	7 (5.0%)	5 (2.9%)	0 (0.0%)	
**Dysfunctional beliefs and attitudes about sleep (DBAS) score**				
<10	10 (6.7%)	3 (1.7%)	1 (2.8%)	*p* = .004
11/20	53 (35.3%)	45 (26.2%)	3 (8.3%)	
21/30	78 (52.0%)	105 (61.0%)	27 (75.0%)	
31/40	9 (6.0%)	19 (11.0%)	5 (13.9%)	

All 36 participants with baseline insomnia completed the immediate (T1) follow-up survey, and 28 (77.8%) completed the follow-up survey at 3 months (T2). The remaining eight had left school (three transferred to other schools, two dropped out for business or marriage, one experienced severe illness, and two were unable to continue due to difficulties paying school fees).

Baseline school environmental characteristics from transect walks and observation notes are summarized in (Table S4).

### Feasibility of the intervention

(1) Sleep education sessions.

There was good fidelity for the sleep education intervention component, with all sessions and topics delivered to teachers, students, and parents. However, not all the sleep education sessions were led by peer educators as intended, as some were delivered by research team leads, or the clinical psychologist, while others were co-facilitated (Table 4). At one school, we conducted an additional session for students and parents as requested by the headteacher, for those who had missed the initial session. Overall, 94% of eligible students (343/366) attended the sleep education session, with lower attendance among parents (n = 151) and teachers and non-teaching school staff (28/99 invited teachers and non-teaching staff). Self-reported attendance data were collected for the 36 participants with baseline insomnia. Of these, 35 (97.2%) reported attending a universal sleep education session, and 24 (66.7%) reported that they and their peers had more knowledge about sleep than they did 6 months ago (i.e. before the intervention).

**Table 4:** Fidelity, dose and reach of the intervention components

	**Fidelity**		**Dose**		**Reach**	
	**Measure**	**Results**	**Measure**	**Results**	**Measure**	Results
**Universal sleep education sessions for students, parents and teachers**	Were all the sleep education topics delivered?	Yes	Proportion of sleep education training topics delivered	100% (5/5)	Number of teachers who attended sleep education session.	28 (of 99) S1^a^: 10 (of 59) S2^a^: 18 (of 40)
	Were the sleep education sessions delivered to teacher, students and parents	Yes	Number of sleep education training sessions delivered to teachers	2 (of 2) S1: 1/1 S2: 1/1	Number of students who attended sleep education session.	343 (of 366) S1: 198 (of 209) S2: 145 (of 155)
	Were all the sleep education sessions delivered as intended by the peer educators	No	Number of sleep education training sessions delivered to students	3 (of 2 intended)^b^ S1: 1 (of 1) S2: 2 (of 1)	Number of parents who attended sleep education session.	151 S1: 63 S2: 88
	Did the project staff track the number of sleep education topics and sessions delivered?	Yes	Number of sleep education training sessions delivered to parents	3 (of 2 intended)^b^ S1: 1 (of 1) S2: 2 (of 1)		
**Universal structural installation of dim bulbs and wall fans**	Were all the proposed installations made?	Yes	Number of dim bulbs and fans installed	S1: 21 dim bulbs, 22 fans S2: 14 dim bulbs, 22 fans	Number of schools that received the structural changes	2 (of 2)
	Were all the installations made in time? (i.e. at baseline)	No^**c**^				
**Universal structural changes to school sleep/wake time**	Were all the proposed adjustments to sleep and wake time implemented?	Yes			Number of schools that implemented the school sleep/wake time changes	2 (of 2)
	Were all the adjustments to sleep/wake time implemented at baseline	No^**c**^			Number of schools that implemented the school sleep/wake time changes at baseline	None (of 2)
	Were all the adjustments to start/finish teaching time implemented at baseline	No^**c**^			Number of schools that adjusted to start/finish teaching time at baseline	None (of 2)
**Group CBT-I for students with baseline insomnias**	Were the CBT-I session groups formed?	Yes	Proportion of CBT-I training sessions delivered	75% (15/15) S1: 10/10 S2: 5/5	Number of participants attending the CBT-I sessions	36 (of 36) S1: 20 S2: 16
	Number of training groups created	3 (of 3) S1: 2 groups S2: 1 group	Number of times the groups met for CBT-I sessions	15 (of 15) S1: 10/10 S2: 5/5	Number of participants who completed all CBT-I sessions	31 (of 36) S1: 18/20 S2: 13/16
	Was the CBT-I implementation tracker completed?	Yes				
	Were all CBT-I topics covered?	Yes				
	Were all the CBT-I sessions delivered by Clinical psychologists?	Yes				

a S1: School one; S2: School two.

b Number of planned sleep education sessions was exceeded after the headteacher requested an additional session for parents and students who had missed the original one.

c The installation of bulbs and fans, as well as the adjustments to school sleep/wake times, were implemented in the second week of October 2024, ~6 weeks after completion of baseline activities in late August, indicating a delay in implementation.

In qualitative interviews, students identified as experiencing insomnia at baseline, teachers and administrators reported that it was feasible to integrate sleep education sessions into existing school subjects or schedules, with teachers indicating increased confidence and preparedness to deliver content following the intervention. School administration support, including headteacher interest, suggests alignment with school priorities and openness to formalizing sleep education (Table 5).

**Table 5:** Qualitative findings on intervention feasibility and acceptability

**Theme**	**Sub-theme**	***Quote***
**Feasibility**	Integrating sleep education sessions into school schedules	*“In the physical education session, there is a topic about rest with a sub-topic about sleep; however, at first I could not exhaust it, but now that I have gained some knowledge, I will be teaching students what I know about sleep.” School 1-IDI- male Teacher* *“I know the headteacher is buying the idea to have sleep education sessions on a given time maybe in a month twice or thrice.” School 2 -IDI-female Teacher*
	Structural Modifications in School Settings	*“We are also constructing extra dormitories and classes, and we intend to install warm lights and fans. Because we also realized that classes are hot, and students are complaining during class time. Day scholars also feel they did not gain much from the study apart from the sleep education session they attended. The structural changes benefited the boarding students, so we are trying to see at least the day scholars also benefit.” School1-IDI-male Teacher*
	Ability to Integrate group CBT-I into Existing Schedules	*“When it came to CBT-I sessions, we used to have them after classes when we were tired, feeling exhausted… it was hard to concentrate.” School 2-IDI-Male student-18 years* *“Having the sessions during class time was better—we were more focused—but it also meant missing lessons, so it needed planning.” School 1-IDI-Female student-16 years*
	CBT-I session duration	*“The CBT-I sessions needed like five hours, and it was given like two to three hours, so more hours or time were needed for the sessions because the facilitators used to use a lot of time” School 2-IDI-Female student 17 years*
**Acceptability**	Content and mode of delivery	*“Some of the technical terms used in the sessions were hard to understand. Using simple English or local languages would help make the information clearer for everyone…” School 2-IDI-Male student 18 years* *“Next time, you could divide the class into small manageable groups so it is easy to track attention and students will understand better…educate each class separately.” School 1-IDI-Female student-16 years* *“… the only difficulty I encountered was the time for completing the diary… Well, you know, school, when you get back, you have to do some laundry, clean up, and more. So, keep in mind you are exhausted for the day and at times you just fall into bed not even recalling that you have to fill in the diary.” School 1- IDI-Female student-15 years* *“Psychologists were confidently talking to us, we asked questions, and they were answered. We could meet them one on one, you tell them your situation, and you would get a solution.” School 2-IDI-Female student 17 years* *“The psychologists were interesting because there were some personal questions you could not ask openly but after the sessions, you could approach one of the facilitators and to ask and also get counseling.” School one IDI-Female student-16 years*
	Peer stigma	*“Students in the dormitory used to talk about me that I have insomnia, so they used to make fun of us who attend the CBT-I sessions.” School 2-IDI-female student 18 years* *“At first, I felt bad because students were like you are the sick people, and you were chosen among us.” School 2-IDI-female student-18 years*
**Effectiveness**	Relevance of the study	*“The study was helpful because some parents do not give time to their children. They are always busy at home, even us here at school, we learnt that we must give these students time to sleep because they are still children,” School 2-IDI-Matron* *“Sleep study needs a lot which students keep demanding, and in a school structure, we cannot provide all of that. For example, the resting time during the day, the students also wanted us to do away with both evening and morning prep.” School 1-IDI-male teacher*
	Intervention effectiveness	*“As teachers, most times we could find students dozing in the daytime, and we could conclude that they are stubborn, and end up even chasing them out of class not knowing they are facing a challenge,” School 2-IDI-female teacher* *“…and what's even more surprising is that I have started to prioritize my own sleep habits after listening to the sessions. I have noticed a huge difference in my own energy levels and focus, and it's made me a better teacher. I wish I had known this earlier" School 1-IDI-male teacher* *“We used to sleep 3 or 2 people on a single- bed with suitcases, but I think our facilitators talked to the administration, and they changed it. Currently, we sleep 1 per bed.” School 1-IDI-male student 17 years* *“They created for us some space for hanging clothes, so the heat was reduced in the dormitory.” School 1-IDI-male student 17 years* *“My experience with a wake-up time is good because when I wake up, I cannot go back to bed again because I have slept enough but before we could sleep wake up, sleep then wake up but nowadays it is okay because we sleep like for 7 to 8 hours.” School one IDI-Female student-16 years* *“Parents were happy with the fans and lights you provided us. When you provided fans, your focus was only on solving the temperature issues in the dormitory, but there is another advantage with fans, and that is chasing away mosquitoes. Mosquitoes cannot be in areas where there are fans, so students sleep well in a very cool environment.” School 1-IDI-male teacher* *"When I acquired knowledge from the sessions, I started respecting sleep and my family did too after sharing what I had learnt. Even my dad, who once saw no importance in it, changed and now gets good rest." School 2-IDI-male student-18 years* *“The container technique. It was last week when I had a problem and I felt like I couldn’t sleep because everything was going wrong, so I put that problem in the container, I put a padlock and sealed it.” School 2-IDI-female student 16 years* *"Before joining the CBT-I sessions, I felt distant and unapproachable, and people saw me as withdrawn and unhappy. But through the sessions, I gained confidence and improved my social interactions.” School 2-IDI-male student 17 years* *“Students in the dormitory used to talk about me that I have insomnia, so they used to make fun of us who attend the CBT-I sessions.” School 2-IDI-female student 18 years* *“At first, I felt bad because students were like you are the sick people, and you were chosen among us.” School 2-IDI-female student-18 years*

(2) Structural changes.

The structural changes were all implemented as intended but not all delivered within the intended timeframe of four weeks before the endline survey (Table 4). Among the 26 boarding students with baseline insomnia, 25 (96.2%) reported noticing changes to the school wake up time with the most reported change being a later school wake up time (n = 17; 68.0%). Almost all (n = 25; 96.2%) reported noticing having new lights that were less bright and having fans installed. About half of participants (n = 12; 46.2%) reported seeing posters about sleep health in school.

These data were supported by the qualitative interviews, which found that the installation of lights and fans was feasible, based on nearly all boarding students reporting them well-received and headteachers committing to install similar dim lights and fans in newly constructed dormitories and classes to support students’ sleep health. Similarly, changes to school sleep times for boarding students were feasible with one school adjusting wake up time for early morning prep from 04:00 to 05:00, and the second school adjusting the wake-up time from 03:00 to 04:30. It was not feasible to alter the official teaching schedule (08:00 to 16:00) due to scheduling constraints and alignment with broader school and national activity calendars (Table 5).

(3) Group CBT-I.

The intended number (five) of CBT-I training sessions were delivered within each group. Of the 36 students with insomnia, 29 (80.6%) reported attending all five sessions, six (16.7%) reported attending four sessions, and one student could not remember how many sessions they attended. The most common reason for not attending was illness. Similarly, the CBT-I implementation tracker found that all 36 participants attended CBT-I sessions (36/36) and 31 (86%) attended all sessions and all the CBT-I planned content was covered (Table 4).

In one school, group CBT-I took place after class hours. Associated challenges related to the fact that many students (particularly day students) were keen to return home, which limited their engagement. Fatigue after a long school day also made it difficult for students to fully absorb the content of the CBT-I sessions, resulting in reduced participation and attentiveness. Time constraints and competing daily responsibilities further hindered students’ ability to consistently attend sessions and complete materials such as the daily sleep diaries (Table 5). In the second school, CBT-I sessions were conducted during class hours, and students were more alert and actively engaged. However, this brought challenges including disruption of academic schedules, requiring careful coordination with school administrators to balance the intervention with regular coursework. In one school with a group CBT-I size of 16 students, facilitators found it challenging to engage all participants and maintain control over discussions. From the interviews with students, groups of 10 students allowed better interaction, deeper engagement, and a more structured learning environment (Table 5).

According to the CBT-I implementation trackers the session durations exceeded the allocated 90-minute time frame, with participants indicating that all sessions ran longer (more than one hour) than scheduled. They also noted that the psychologists needed an additional hour to adequately cover the session materials, address participant questions and concerns, or provide sufficient support and guidance. The extended session durations also reflected the complexity of the participants' sleep issues and the need for more personalized attention.

### Acceptability of the intervention

Students and teachers provided positive feedback on both universal and targeted components, highlighting the content as engaging, informative, clear, and well organized. Sleep education, structural changes, and CBT-I improved students’ sleep habits, awareness, and well-being, and positively influenced teachers’ attitudes and sleep practices at school. Challenges included peer stigma, environmental discomfort, and adherence difficulties, particularly among day students. (Table 5).

(1) Sleep education sessions.

Many students expressed a preference for sessions to be delivered in smaller groups (by individual class), citing difficulty hearing the facilitators over background noise in the large group sessions (~150–180 students per session). Teachers and parents found sleep education sessions appropriate and potentially helpful for their students, especially since parents tend not to help their children manage sleep-related challenges (Table 5).

Students gained a better understanding of sleep’s importance, dispelling misconceptions and adopting consistent bedtime routines (Table 5). Teachers previously misinterpreted daytime sleepiness as disobedience, often leading to punishment. The intervention improved their awareness, fostering a supportive environment that enhanced students’ sleep habits. Both teachers and students reported increased knowledge and motivation to prioritize sleep, contributing to a more sleep-conscious school culture.

(2) Structural intervention.

Students reported fans improved sleep by reducing heat and deterring mosquitoes, while dim lights and adjusted study schedules promoted earlier bedtimes and consistent wake-up routines. Challenges included fan misuse (running unnecessarily, using newly added sockets to power irons), lighting and heat issues, and teacher resistance to timetable changes due to academic concerns. Teachers expressed that altering school start times could negatively impact performance and deemed some structural requests unfeasible. Students recommended enforcing dormitory rules, regular maintenance of fans and lights, and responsible facility use. These adjustments, they noted, would enhance sleep quality and maximize program benefits.

(3) Group CBT-I.

Students valued receiving CBT-I from trained psychologists, appreciating their engaging, relatable, and non-judgmental approach. The warm, interactive style fostered trust and encouraged open discussions about sleep challenges. They benefited from learning and applying tailored strategies such as sleep restriction, stimulus control, relaxation techniques, and consistent bed/wake times.

Structured sessions, featuring guided exercises, pictorial coloring, journaling, and group discussions, helped students understand sleep hygiene (limiting screen time, reducing caffeine, creating bedtime routines) and manage challenges like racing thoughts and irregular sleep schedules. Daily sleep diaries enabled progress tracking and ownership of sleep habits. Participants reported increased confidence in managing their sleep, improved peer interactions due to reduced fatigue and irritability, and positive changes in household routines such as younger siblings adopting better sleep habits. However, some found technical terms like 'stimulus control’ or “cognitive restructuring” difficult to understand and suggested using simpler language or a mix of English and Luganda. Day students recommended weekend sessions to avoid school conflicts. A few reported peer stigma, being labeled “sleep patients,” highlighting the need for greater awareness to reduce misconceptions.

### Indicative impacts of the intervention on sleep and mental health among students with baseline insomnia

Among students with baseline insomnia, there was strong evidence of a decrease in mean ISI score from T0 to T1 (16.83 vs 9.81, *p* < .001), with 15 (41.7%) of participants reporting no insomnia symptoms at T1. Improvements were sustained at T2 (Table 6). Most participants had a *>* 6 point reduction in the ISI score (deemed to represent a clinically meaningful improvement [39]) at both T1 (29/36; 69.4%) and T2 (22/28; 78.6%). There was also strong evidence of a decrease in depression and anxiety symptoms during follow-up (Table 6).

**Table 6:** Sleep characteristics at baseline, 6-week and 3-month follow-up among sub-group with insomnia

	**Baseline (T0)**	**6-week follow-up (T1)**	**3 month follow-up (T2)**	***p*-value (T1 vs T0)**	***p*-value (T2 vs T0)**
**N (%)**	**36 (100.0%)**	**36 (100.0%)**	**28 (77.8%)**		
**Insomnia**					
**Insomnia Severity Index Score (mean (SE))**	16.83 (2.04)	9.81 (5.60)	7.14 (4.75)	<0.001	<0.001
**Insomnia Severity Index Category**					
No clinical insomnia (0/7)	0 (0.0%)	15 (41.7%)	14 (50.0%)	<0.001	<0.001
Subthreshold insomnia (8/14)	0 (0.0%)	14 (38.9%)	13 (46.4%)		
Moderate clinical insomnia (15/21)	35 (97.2%)	7 (19.4%)	1 (3.6%)		
Severe clinical insomnia (22/28)	1 (2.8%)	0 (0.0%)	0 (0.0%)		
**Proportion with a decrease of *>* 6 points on ISI score from baseline**	n/a	29 (69.4%)	22 (78.6%)	n/a	n/a
**Mental health**					
**PHQ-9 total score (mean (SD))**	11.94 (4.34)	8.92 (5.22)	8.32 (4.38)	0.001	<0.001
**PHQ-9 category**					
No depression (0-4)	0 (0.0%)	8 (22.2%)	5 (17.9%)	0.006	0.001
Mild depression (5-9)	13 (36.1%)	13 (36.1%)	13 (46.4%)		
Moderate depression (10-14)	12 (33.3%)	8 (22.2%)	7 (25.0%)		
Moderately severe depression (15-19)	9 (25.0%)	6 (16.7%)	3 (10.7%)		
Severe depression (20-27)	2 (5.6%)	1 (2.8%)	0 (0.0%)		
**GAD-7 total score (mean (SD))**	8.97 (3.92)	6.75 (5.04)	5.86 (3.69)	0.01	<0.001
**GAD-7 category**					
Minimal anxiety (0-4)	3 (8.3%)	14 (38.9%)	10 (35.7%)	0.02	0.002
Mild anxiety (5-9)	18 (50.0%)	11 (30.6%)	14 (50.0%)		
Moderate anxiety (10-14)	9 (25.0%)	7 (19.4%)	3 (10.7%)		
Severe anxiety (15-21)	6 (16.7%)	4 (11.1%)	1 (3.6%)		
**Main reason for poor sleep**					
**What is the most common reason you typically have a poor night sleep?**					
It is too noisy	5 (13.9%)	7 (20.6%)	4 (16.0%)		
It is too light	1 (2.8%)	2 (5.9%)	0 (0.0%)		
It is too hot/cold	4 (11.1%)	4 (11.8%)	7 (28.0%)		
I am worried or anxious	6 (16.7%)	3 (8.8%)	2 (8.0%)		
I am in pain	9 (25.0%)	7 (20.6%)	4 (16.0%)		
Heavy school timetable	11 (30.6%)	9 (26.5%)	7 (28.0%)		
Other	0 (0.0%)	2 (5.9%)	1 (4.0%)		
**Sleepiness**					
I go through the whole school day without feeling tired					
Never/rarely/sometimes	20 (55.6%)	25 (69.4%)	21 (75.0%)	0.23	0.13
Often/always	16 (44.4%)	11 (30.6%)	7 (25.0%)		
I fall asleep during my morning classes					
Never/rarely/sometimes	24 (66.7%)	28 (77.8%)	25 (89.3%)	0.32	0.03
Often/always	12 (33.3%)	8 (22.2%)	3 (10.7%)		
I fall asleep during the last class of the day					
Never/rarely/sometimes	26 (72.2%)	28 (77.8%)	25 (89.3%)	0.48	0.10
Often/always	10 (27.8%)	8 (22.2%)	3 (10.7%)		
I fall asleep at school in my afternoon classes					
Never/rarely/sometimes	24 (66.7%)	30 (83.3%)	25 (89.3%)	0.08	0.16
Often/always	12 (33.3%)	6 (16.7%)	3 (10.7%)		
During the school day, there are times when I realize that I have just fallen asleep					
Never/rarely/sometimes	26 (72.2%)	30 (83.3%)	28 (100%)	0.25	0.005
Often/always	10 (27.8%)	6 (16.7%)	0 (0.0%)		
**School day sleep characteristics**					
**Sleep duration (mean SD)**	4.02 (1.47)	6.41 (1.43)	6.13 (1.56)	<0.001	<0.001
**Sleep duration**					
<7 hours	35 (97.2%)	26 (72.2%)	20 (71.4%)	0.03	0.05
7-11 hours	1 (2.8%)	10 (27.8%)	8 (28.6%)		
> = 12 hours	0 (0%)	0 (0%)	0 (0%)		
**Total time in bed (mean (SD))**	5.84 (1.39)	7.46 (1.32)	6.99 (1.48)	<0.001	<0.001
**Total time in bed**					
<7 hours	29 (80.6%)	15 (41.7%)	13 (46.4%)	<0.001	0.03
7-11 hours	7 (19.4%)	20 (55.6%)	14 (50.0%)		
> = 12 hours	0 (0.0%)	1 (2.8%)	1 (3.6%)		
**Sleep efficiency**					
*>*85% (good)	8 (22.2%)	19 (52.8%)	18 (64.3%)	0.02	0.01
<85% (poor)	28 (77.8%)	17 (47.2%)	10 (35.7%)		
**Non-school days**					
**Sleep duration (mean (SD))**	5.43 (2.52)	6.57 (2.09)	6.64 (2.82)	0.002	0.04
**Sleep duration**					
<7 hours	27 (75.0%)	24 (66.7%)	15 (53.6%)	0.33	0.19
7-11 hours	8 (22.2%)	12 (33.3%)	13 (46.4%)		
> = 12 hours	1 (2.8%)	0 (0.0%)	0 (0.0%)		
**Total time in bed (mean (SD))**	6.60 (2.60)	7.70 (2.11)	7.40 (2.77)	0.02	0.14
**Total time in bed**					
<7 hours	24 (66.7%)	14 (38.9%)	9 (32.1%)	0.005	0.02
7-11 hours	10 (27.8%)	22 (61.1%)	19 (67.9%)		
> = 12 hours	2 (5.6%)	0 (0.0%)	0 (0.0%)		
**Sleep efficiency**					
*>*85% (good)	19 (52.8%)	24 (66.7%)	21 (75.0%)	0.26	0.07
<85% (poor)	17 (47.2%)	12 (33.3%)	7 (25.0%)		
**Chronotype**					
<3 a.m. (early)	9 (25.0%)	15 (41.7%)	19 (67.9%)	0.14	0.01
> = 3 a.m. (late)	27 (75.0%)	21 (58.3%)	9 (32.1%)		
**DBAS score (mean (SD))**	25.72 (5.82)	22.36 (6.7)	23.57 (7.3)	0.005	0.15
**DBAS category**					
Few dysfunctional beliefs and attitudes (<10)	1 (2.8%)	1 (2.8%)	1 (3.6%)	0.002	0.12
11/20	3 (8.3%)	13 (36.1%)	8 (28.6%)		
21/30	27 (75.0%)	18 (50.0%)	15 (53.6%)		
Many dysfunctional beliefs and attitudes (31/40)	5 (13.9%)	4 (11.1%)	4 (14.3%)		

**Figure 1:**
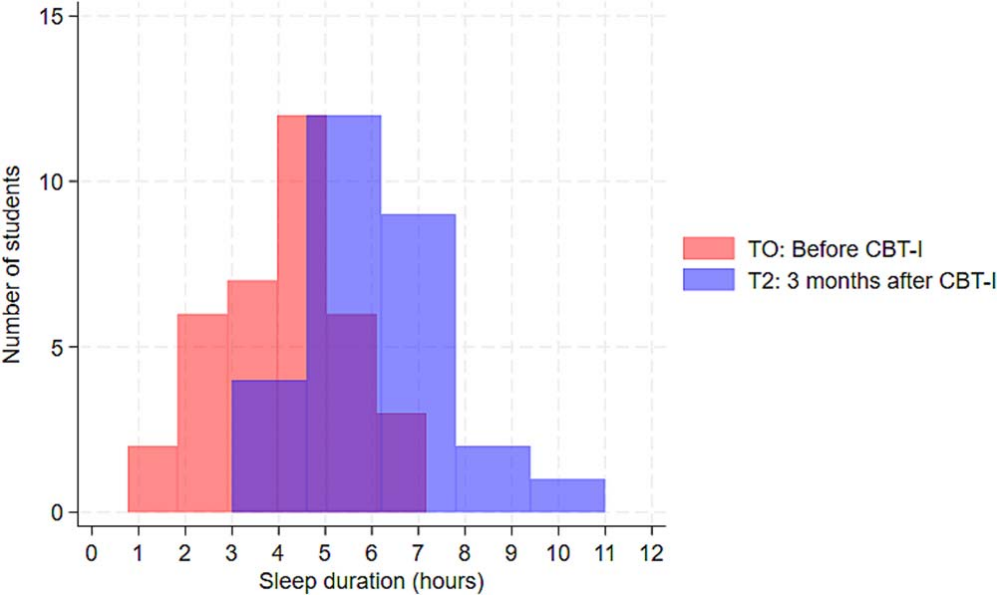
Change in sleep duration on schooldays at baseline (T0) and 3 months after CBT-I (T2) among students with moderate/severe insomnia (CBT-I subgroup).

The mean sleep duration during school days increased from 4.02 to 6.13 hours at T2 (*p* < .001; Figure 1), as did total TIB (from 5.84 to 6.99 hours at T2; *p* < .001) and proportion with *>*85% sleep efficiency (from 22.2% to 64.3%; *p* = .01). There was similar indicative evidence of improvements on non-school days. Averaged over the week, students shifted to an earlier chronotype at T2 compared to baseline (from 25.0% at T0 to 67.9% at T2: *p* = .01), and the DBAS score (reflecting dysfunctional beliefs about sleep) showed a small, non-significant reduction from 25.72 to 23.57 at T2 (*p* = .15) (Table 6).

Survey data showed an improvement in sleep-related knowledge and goal setting by Week 5 of CBT-I (Table S5). The sleep diary data also showed indicative evidence of improvements in sleep health and related behaviours (Table S5).

## Discussion

The study showed that a school-based, tiered sleep health intervention was feasible and acceptable among Ugandan adolescents, with engagement and successful integration into school schedules. Overall, there was strong fidelity, except for the intended 90 minute duration of CBT-I groups, and most participants received the intended dose. Structural changes, such as improved lighting and study schedules, were well-implemented, though start time adjustments varied between the schools. Students, parents, and teachers viewed the intervention as beneficial, though concerns about sustainability and stigma around CBT-I attendance were noted. Despite logistical challenges in delivering education and therapy sessions, most participants received the intended dose. The intervention showed potential for clinically meaningful improvements in insomnia, sleep opportunity (total time in bed), sleep health, depression, and anxiety, supporting its scalability in similar settings.

The universal sleep education component was aided by strong school leadership and integration into existing schedules resulting in high student uptake. However, participation from teachers and parents was lower, likely reflecting competing responsibilities and limited prioritization of sleep health. This aligns with findings from previous school-based sleep education studies, that while students engage readily, adult involvement is hindered by time constraints, low prioritization of sleep health, and limited awareness of its importance [40, 41]. Involving parents and teachers is potentially important, as they can help reinforce sleep-promoting behaviours and create consistent, and supportive environments at home and school, enhancing the effectiveness and sustainability of interventions [42]. To improve parent and teacher engagement, targeted sleep heath intervention for example, providing tailored information sessions, workshops, or materials that address parents’ and teachers’ specific roles in supporting adolescent sleep together and scheduling flexibility may be essential.

The CBT-I and education sessions were well accepted, reflecting content relevance. However, smaller group sizes are recommended, as classroom-based formats with 20–30 students enhance engagement, interaction, and personalized feedback compared to larger groups [43].

Teachers reported understanding adolescent sleep needs better due to the education sessions, suggesting that sleep education can shift adult perceptions when they understand sleep deprivation as a health, not behavioral issue. This supports findings from a Dutch study showing improved teacher attitudes towards sleep, and behavioral improvements of students, after school-based a sleep education interventions [44]. In terms of effectiveness, we found preliminary evidence of improvements in sleep knowledge, hygiene practices, and teacher responses to sleep-related challenges. These results align with the SIESTA intervention in Scotland [40] and the Healthy Sleep for Healthy Schools (HS4HS) study in Australia [45], although other studies suggest that more intensive or personalized approaches may be required for lasting behavioral change [46].

To our knowledge, this is the first study to evaluate the feasibility and acceptability of undertaking physical environmental changes to light, temperature and airflow in boarding schools, and the first in Africa to implement adjustments to school wake-up times. Some delays in the installation of fans occurred, but schools expressed commitment to maintaining the changes and incorporating sleep-friendly features in future. Previous studies on delayed school start times have shown that shifting schedules by 30–60 minutes leads to longer sleep duration, improved mood, concentration, academic performance, better attendance, reduced daytime sleepiness and tardiness, and fewer teen driving accidents, especially among adolescents with later chronotypes [47, 48]. However, schools in the UK resisted proposed delays to school start times suggested by researchers due to the need to align with national academic schedules, and similar findings have been seen in the US, where changes to start times are often constrained by parental preferences and logistical factors [49, 50].

The CBT-I component was implemented with good fidelity, despite scheduling challenges and larger groups than anticipated. This was primarily due to the limited time schools allowed for CBT-I delivery, which required combining more students into each session especially in school two. Students were strongly engaged with the sessions and described them as helpful and relevant. This suggests that school-based group CBT-I can be both culturally adaptable and practically feasible in low-resource settings, supporting similar evidence from a study conducted in Nigeria [18] and South Africa [17].

Some students reported experiencing stigma from peers related to attending the CBT-I sessions. Notably, this occurred despite all students in the selected year groups receiving universal sleep education. This indicates that awareness alone may not reduce stigma and can sometimes reinforce perceptions of sleep difficulties as pathological, leading to labeling or “us vs. them” dynamics. Future interventions should combine education with nuanced framing and peer-led discussions to normalize sleep challenges without creating new stigmatising categories [51].

In terms of preliminary effectiveness, students reported substantial reductions in insomnia symptoms and comorbid anxiety and depression, consistent with findings from randomized trials in high-income adolescent populations [9, 12, 13, 52]. Improvements in sleep knowledge, total time in bed, longer sleep duration, and healthier attitudes reflect durable cognitive and behavioral change [53, 54]. The current CBT-I delivery model using external psychologists may not be scalable in low-resource schools. Future research should explore more sustainable approaches, such as training school staff or peers to deliver CBT-I, to enable broader implementation and lasting impact.

Strengths of the study include its use of a tiered intervention, incorporating both universal sleep health education and targeted CBT-I for adolescents screening positive for moderate/severe insomnia, providing comprehensive demographic coverage. The mixed-methods approach (qualitative and quantitative) enabled robust assessment of feasibility and acceptability. Longitudinal follow-up of those with insomnia allowed for measurement of the intervention’s indicative impact. However, the absence of follow-up data for students without baseline insomnia and limitation to only two schools within a single district reduce generalizability. Without a control group, improvements in sleep and mental health symptoms cannot be confidently attributed to the intervention, as they may reflect regression to the mean or unrelated external factors. In addition, social and structural factors beyond the intervention may have influenced sleep outcomes, including day students’ home living arrangements, dormitory conditions (e.g. crowding, comfort, or sleep-disrupting pain), and broader contextual or policy-level factors, such as school schedules, community noise, or changes in educational policies. These factors should be considered when planning future interventions. Monitoring fidelity and reach via session logs and attendance registers may not adequately capture nuances in intervention quality or actual participant engagement. We did not assess inter-rater reliability for fidelity ratings, and this may have reduced their validity, although we mitigated this by having a pair of team members conduct the assessment and agree ratings through discussion at the time of assessment.

## Conclusion

This study found a school-based, tiered sleep health intervention featuring culturally adapted group CBT-I to be feasible and acceptable in a low-resource Ugandan context, delivered with high fidelity and strong student engagement. Improvements were seen in insomnia, sleep behaviors, and mental health outcomes. Key barriers included limited teacher and parent involvement, relatively inflexible school schedules, and peer stigma, highlighting the importance of whole-school engagement. These findings add evidence that multi-level, school-based sleep interventions can be successful in low-income settings. Large-scale cluster-randomized controlled trials are needed to estimate impact and cost-effectiveness. Future efforts should emphasize sustainability planning and stigma reduction to maximize long-term benefits and improve adolescent wellbeing through context-sensitive delivery and greater institutional support.

## Supplementary Material

Supplementary_Tables_Clean_zpag029

## Data Availability

The data are available on request from LSHTM Data Compass.

## Notes

Trial Name: Better sleep, better health: designing a school based intervention in Uganda. URL: https://doi.org/10.1186/ISRCTN55077169

Trial registration: ISRCTN 55077169, Registered April 06, 2025, retrospectively registered.
