# Supplementary material for: Feasibility and acceptability of a sleep health intervention among adolescents in Ugandan schools: A prospective pilot intervention study
Source: Sleep Adv. 2026 Mar 9;7(2):zpag029. doi: 10.1093/sleepadvances/zpag029 (PMC13109094; doi:10.1093/sleepadvances/zpag029)
Supplement: Supplementary_Tables_Clean_zpag029 [file supplementary_tables_clean_zpag029.docx]

**Title: Feasibility and acceptability of a sleep health intervention among adolescents in Ugandan schools: A prospective pilot intervention study.**

**Authors**

Denis Ndekezi^1,2^, Rebecca Kyomugisha^2^ Betty Nyangoma^2^, Prossy Namirembe^2^, Beatrice Nanyonga^2^, Aaron Nyaruhuma^3^, Claudia Ateo^3^, Calvin Robert Rutainama^3^, Katherine A Thomas^4^, Ratifah Batuusa^2^, Benson Muhindo^3^, Sheilah Kasabiiti^3^, Connie Alezuyo^5^, Nambusi Kyegombe^2,6^, Chris Bonell^7^, Daniel Michelson^8,9^, Fiona C. Baker^10^, Faith Orchard^11^, Femke Bannink Mbazzi^1,2^, Helen A Weiss^4^

**Affiliations**

1. Department of Population Health, London School of Hygiene and Tropical Medicine
2. MRC/UVRI and LSHTM Uganda Research Unit, Entebbe, Uganda.
3. Reach A Hand Uganda
4. International Statistics and Epidemiology Group, London School of Hygiene & Tropical Medicine, London, United Kingdom
5. Ministry of Education and Sports, Uganda.
6. Department of Global Health and Development, London School of Hygiene and Tropical Medicine
7. Department of Public Health, Environments and Society, London School of Hygiene & Tropical Medicine, London, UK
8. Dept of Child & Adolescent Psychiatry, Institute of Psychiatry, Psychology & Neuroscience, King’s College London, London, UK
9. NIHR Maudsley Biomedical Research Centre, South London and Maudsley NHS Foundation Trust and King’s College London, London, UK
10. Center for Health Sciences, SRI International, Menlo Park, CA, USA and School of Physiology, University of the Witwatersrand, Johannesburg, South Africa
11. School of Psychology, University of Sussex, Sussex, UK

**Corresponding author**: Denis Ndekezi ([Denis.Ndekezi@lshtm.ac.uk](mailto:Denis.Ndekezi@lshtm.ac.uk) )

**Table S1: Measures of sleep health and insomnia**

| Sleep Health measure | Tool | Question | Range & interpretation |
| --- | --- | --- | --- |
| Satisfaction | Insomnia Severity Index^a^ | How satisfied/dissatisfied are you with your current sleep pattern (in the past month)? | 5 point Likert scale converted to binary  (Moderately satisfied, dissatisfied, or very dissatisfied) vs (Very satisfied or satisfied) |
|  |  | How worried/distressed are you about your current sleep? | 5 point Likert scale converted to binary  (Very much or much worried) vs (Somewhat, a little or not at all worried) |
| Alertness/Sleepiness | Cleveland Adolescent Sleepiness Questionnaire | I go through the whole day without feeling tired | 5 point Likert scale scored from 1-5, with 1 being least sleepy to 5 being most sleepy.  The CSAQ score was defined as the sum of the 5 item scores and binarized with a cut-off of 10. This cut-off corresponds to an average reporting of never or rarely feeling sleepy on each item. |
|  |  | I fall asleep during morning class |  |
|  |  | I fall asleep during the last class of the day |  |
|  |  | I fall asleep in my afternoon classes |  |
|  |  | During the school day, there are times when I realize that I have just fallen asleep |  |
| Sleep timing | Munich ChronoType Questionnaire | I go to bed at…  I actually get ready to go to sleep at…  I need … minutes to fall asleep  I wake up at….  After … minutes I get up | Chronotype calculated as the midpoint of sleep, and categorised as before or after 2am |
| Sleep efficiency |  |  | Proportion of time in bed spent asleep, and categorised as <85% or >85% |
| Sleep duration |  |  | Total time in bed |

^a^ These are the two items related to satisfaction. All 7 items of the Insomnia Severity Index were included in the survey.

**Table S2: The UNICEF Measuring Mental Health Among Adolescents and Young People at the Population Level (MMAP tool)**

| Item | Question: During the past two weeks, how often have … | Included in |
| --- | --- | --- |
| 1 | you been feeling very sad or depressed? | Depression screening and score |
| 2 | you felt easily annoyed or angry at small things? |  |
| 3 | you not enjoyed doing things you used to enjoy, such as playing sports, singing and dancing, spending time with friends, or watching videos? |  |
| 4 | you felt hopeless about the future? |  |
| 5 | you felt nervous, anxious, or on edge? | Anxiety screening and score |
| 6 | you worried you can’t do anything right or are doing things poorly? |  |
| 7 | you worried about what others think of you? |  |
| 8 | you worried something bad will happen to you or your family? |  |
| 9 | you worried too much about different things? |  |
| 10 | you felt unable to stop or control your worries? |  |
| 11 | you not wanted to eat even when food was available, or have you eaten too much? | Depression score |
| 12 | you had problems falling asleep, problems sleeping well, or problems with sleeping too much? |  |
| 13 | you felt that you got tired easily or did not have the energy to do daily activities? |  |
| 14 | you had trouble concentrating on things, such as doing homework, household chores, or other activities, such as watching videos or using social media, for example, WhatsApp or Instagram? |  |
| 15 | you felt lonely? |  |
| 16 | you felt like a failure or like you have let yourself or your family down? |  |
| 17 | you had thoughts that you would rather be dead or thoughts of hurting yourself? |  |
| 18 | others said that you have been moving more slowly than usual? |  |
| 19 | others said that you are restless or that you can’t sit still? | Depression and anxiety  scores |
| 20 | you felt that it was difficult to breathe? |  |
| 21 | you felt dizzy or faint? |  |
| 22 | you suddenly got scared for no reason or without knowing what made you scared? | Anxiety score |
| 23 | you had difficulty relaxing or difficulty feeling calm? |  |
| 24 | you felt like your heart was pounding or beating too fast? |  |
| 25 | you had headaches or muscle tension? |  |

**Table S3: Adaptation of Group CBT-I for Ugandan Adolescents: Comparison with the Nigerian Trial.**

| **Domain** | **Nigeria (Egbegi et al., 2021)** | **Uganda (current study adaptation)** | **Rationale for Ugandan adaptation** |
| --- | --- | --- | --- |
| **Core intervention** | Manualised group CBT-I, 5 weekly sessions (45 min each), | Retained manualised group CBT-I, 5 weekly sessions (45–60 min) in school settings. | To maintain fidelity to evidence-based CBT-I while ensuring feasibility in Ugandan schools. |
| **Delivery** | Delivered by counselling psychologist (no formal CBT-I certification) under weekly supervision. | Delivered by licensed clinical psychologists trained in adolescent mental health and CBT, supervised by a senior clinical psychologist. | To strengthen fidelity, acceptability, and professional oversight in the Ugandan context. |
| **Sleep diaries / logs** | Sleep logs avoided to reduce burden. | Used simplified daily **sleep diaries** to track sleep patterns and aid reflection. | Diaries were feasible in Ugandan schools and provided useful monitoring without heavy burden. |
| **Stimulus control (bed use)** | Bedroom use allowed for schoolwork due to limited space. | Adolescents encouraged to **remove items from the bed** (books, phones) and use the bed only for sleep. | More practical in Ugandan boarding schools where dormitories had space constraints, but beds could still be restricted to sleep use. |
| **Leaving bed after 15–20 min awake** | Not encouraged, as lack of space/parental restrictions made it impractical. | Also not encouraged; instead, relaxation techniques (breathing, muscle relaxation, imagery) practiced while in bed. | Boarding school dormitories made “leaving bed” unrealistic; relaxation was a culturally and structurally acceptable substitute. |
| **Sleep restriction** | Limited to discouraging daytime naps (full bedtime restriction omitted). | Same modification applied (focus on reducing long daytime naps). | Daytime napping was a more feasible and contextually acceptable target than strict sleep restriction. |
| **Language of delivery** | English (language of instruction; fluent among participants). | English used, with culturally relevant examples integrated. | To ensure comprehension and resonance with Ugandan adolescents. |
| **Feasibility considerations** | Sessions during school breaks; sensitivity to parental concerns about interfering with academics. | Sessions delivered at times agreed with school administrators: in one school after lunch break, in another after school in the evening. | To align with school schedules, minimise disruption, and build administrative support. |

**Table S4. Baseline school environmental and dormitory characteristics**

| **Domain** | **School 1** | **School 2** |
| --- | --- | --- |
| **School type and layout** | Mixed day/boarding secondary school located on a small piece of land with closely-spaced structures and a limited compound. No designated resting or shaded areas. | Mixed day/boarding secondary school located on a small land area, but relatively larger than School 1, with congested structures and a restricted compound. No shaded areas; spatial organisation was slightly less congested than School 1. |
| **Dormitory access and management** | Boarding students not allowed to access dormitories during the day; dormitories closed from 6am to 5pm. Dormitories managed by student captains under supervision of a warden (boys) and matron (girls). | |
| **Dormitory number, location, and privacy** | Boys: four dormitories (not class-specific). Girls: 3 dormitories organized by class level. Dormitories located behind classrooms, providing some degree of privacy. | Boys: two dormitories organized by class level. Girls: three dormitories organized by class level. located at the front of the main compound adjacent to the main hall; girls reported limited privacy. |
| **Sleeping arrangements and congestion** | Wooden triple-decker bunk beds, some old. Congestion observed in most dormitories; limited spacing between beds (except in one girls’ dormitory). Students often slept with metallic suitcases on beds, reducing sleeping space. | Metallic triple-decker bunk beds. Congestion observed but generally less pronounced than in School 1, with relatively more space between beds and fewer obstructions on beds. |
| **Physical sleep environment (ventilation, lighting, temperature)** | Poor ventilation observed in girls’ dormitories, with restricted airflow and stuffy conditions without any mechanical support. No fans were available to support air circulation. The poor ventilation observed in School 1 reflected restricted air flow and stuffy dormitory conditions without any mechanical support.  Lighting was primarily provided by long overhead fluorescents tube fixtures mounted on the timber roofs, producing bright illumination but with limited control, as some switches were located outside dormitories. No lamps were available. In School 1, switches were located outside dormitories, limiting night-time control.  Iron-sheet roofing contributed to high indoor temperatures; teachers reported excessive classroom heat. | Ventilation, lighting, and thermal conditions observed to be generally better than in School 1, with improved airflow. No fans were present, but switches for overhead fluorescent tube lights were located inside dormitories, allowing better night-time control. No lamps were available.  Indoor temperatures were similar but appeared slightly moderated by better ventilation. |
| **Dormitory wake times and teaching schedules.** | Students wake at 03:00, with 30 minutes allocated for preparation before attending morning Muslim prayers (30 minutes). At 04:00, they begin morning preps, which continue until 07:30. Formal classes start at 08:00, with a mid-morning break from 10:30–11:00. Classes resume until 13:00, followed by a one-hour lunch break. Afternoon classes run from 14:00–17:00, after which day students return home. Boarding students engage in co-curricular activities and personal preparation before evening preps, held from 19:00–21:30. | Students wake at 04:00, with 30 minutes for preparation before starting morning preps at 04:30. Morning preps end at 07:30, followed by formal classes beginning at 08:00. The remainder of the daily schedule mirrors School 1, including the mid-morning break, lunch, afternoon classes, and evening preps. |
| **Cleanliness and general condition** | Dormitories generally clean. | Dormitories generally clean and maintained with a cleaning rota. |
| **Mosquito net use** | Limited use of mosquito nets observed. | Not assessed. |
| **Health and rest facilities** | Sick bay available for girls only; no designated daytime resting space for boys. | Sick bay available; no designated daytime resting spaces for boarding students. |

**Table S5: Self-reported sleep characteristics pre- and post group CBT-I among 36 students receiving group CBT-I**

| **N** | **Week 1** | **Week 5** | **Odds ratio (95%CI)** | | **p-value** |
| --- | --- | --- | --- | --- | --- |
| **Sleep health characteristics** | N=36 | N=36 |  |  | |
| Total sleep time (hours) (median, IQR) | 6.36  (5.00-7.33) | 6.67  (5.75-7.58) | 0.36 (-0.03, 0.74) | 0.03 | |
| Time going to sleep (median, IQR) | 11pm  (10.12-11.48) | 10.25pm  (10.00-11.00) |  | <0.001 | |
| Time getting up (median, IQR) | 5am  (4.15-6.00) | 5.05am  (4.50-6.00) |  | 0.72 | |
| % of nights with 7-11 hours sleep | 79 (31.4%) | 106 (42.1%) | 1.59 (1.10-2.29) | 0.01 | |
| % of days woke up feeling rested | 73 (29.7%) | 128 (51.6%) | 2.53 (1.75-3.66) | <0.001 | |
| % reporting no chance of falling asleep while studying | 75 (30.0%) | 96 (38.7%) | 1.47 (1.02, 2.14) | 0.04 | |
| % of days napped during the day | 160 (63.8%) | 132 (52.4%) | 0.63 (0.44-0.89) | 0.01 | |
| **% of nights feel asleep:** |  |  |  |  | |
| Easily | 100 (39.8%) | 105 (42.7%) | 1 | 0.01 | |
| Took some time | 104 (41.4%) | 118 (48.0%) | 1.08 (0.74-1.58) |  | |
| With difficulty | 47 (18.7%) | 23 (9.4%) | 0.47 (0.26-0.82) |  | |
| **% with sleep disturbed by:** |  |  |  |  | |
| Noise | 83 (32.9%) | 56 (22.2%) | 0.58 (0.39-0.86) | 0.007 | |
| Light | 80 (31.8%) | 20 (7.9%) | 0.19 (0.11-0.31) | <0.001 | |
| Temperature | 49 (19.4%) | 17 (6.8%) | 0.30 (0.17-0.54) | <0.001 | |
| Nightmares | 10 (4.0%) | 17 (6.8%) | 1.75 (0.78-3.90) | 0.17 | |
| Discomfort | 51 (20.2%) | 20 (7.9%) | 0.34 (0.20-0.59) | <0.001 | |
| Stress | 45 (17.9%) | 32 (12.7%) | 0.67 (0.41-1.09) | 0.11 | |
| Pain | 58 (23.0%) | 36 (14.3%) | 0.56 (0.35-0.88) | 0.01 | |
| Not disturbed | 45 (17.9%) | 82 (32.5%) | 2.22 (1.46-3.37) | <0.001 | |
| **Sleep behavior characteristics** |  |  |  |  | |
| **% with bedtime routine including:** |  |  |  |  | |
| Reading | 83 (32.9%) | 109 (43.3%) | 1.55 (1.09-2.23) | 0.02 | |
| Bathing | 109 (43.3%) | 107 (42.5%) | 0.97 (0.68-1.38) | 0.86 | |
| Studying | 45 (17.9%) | 40 (15.9%) | 0.87 (0.54-1.38) | 0.55 | |
| Relaxation exercise | 55 (21.8%) | 70 (27.8%) | 1.38 (0.92-2.07) | 0.12 | |
| Using electronics | 65 (25.8%) | 50 (19.8%) | 0.71 (0.47-1.08) | 0.11 | |
| % of days consumed caffeine in the evening | 72 (28.6%) | 54 (21.4%) | 0.68 (0.45-1.02) | 0.07 | |
| % of days exercised at least 20 minutes in the: |  |  |  |  | |
| Morning | 62 (24.6%) | 67 (26.6%) | 1.11 (0.74-1.66) | 0.61 | |
| Afternoon | 21 (8.3%) | 53 (21.0%) | 2.93 (1.71-5.03) | <0.001 | |
| Evening | 88 (34.9%) | 100 (39.7%) | 1.23 (0.85-1.76) | 0.27 | |
|  |  |  |  |  | |
| **% of days when mood is** |  |  |  |  | |
| Very Poor |  |  |  |  | |
| Poor | 45 (17.9%) | 16 (6.5%) | 1 | <0.001 | |
| Good | 51 (20.2%) | 34 (13.7%) | 1.87 (0.92-3.84) |  | |
| Very good | 101 (40.1%) | 122 (49.9%) | 3.40 (1.81-6.37) |  | |
